# Detection of preanalytical errors in arterial blood gas analysis

**DOI:** 10.11613/BM.2022.020708

**Published:** 2022-06-15

**Authors:** Serap Çuhadar, Hayat Özkanay-Yörük, Mehmet Köseoğlu, Kaan Katırcıoğlu

**Affiliations:** 1 Department of Biochemistry, Atatürk Training and Research Hospital, Izmir, Turkey; 2 Department of Biochemistry, Tınaztepe University, Izmir, Turkey; 3 Department of Anesthesiology and Reanimation, Tınaztepe University, Izmir, Turkey

**Keywords:** blood-gas analysis, plastic syringe, pO_2_, pCO_2_, preanalytical variables

## Abstract

**Introduction:**

Blood gas analysis (BGA) is an essential test used for years to provide vital information in critically ill patients. However, the instability of the blood gases is a problem. We aimed to evaluate time and temperature effects on blood gas stability.

**Materials and methods:**

Arterial blood was collected from 20 patients into syringes. Following BGA for baseline, syringes were divided into groups to stand at 4°C and 22°C for 30, 60, 90, 120 minutes. All were tested for pH, partial pressure of carbon dioxide (pCO_2_), partial pressure of oxygen (pO_2_), oxygen saturation (sO_2_), oxyhemoglobin (O_2_Hb), sodium, potassium, glucose, lactate, oxygen tension at 50% hemoglobin saturation (p50), and bicarbonate. A subgroup analysis was performed to detect the effect of air on results during storage. Percentage deviations were calculated and compared against the preset quality specifications for allowable total error.

**Results:**

At 4°C, pO_2_ was the least stable parameter. At 22°C, pO_2_ remained stable for 120 min, pH and glucose for 90 min, lactate and pCO_2_ for 60 min. Glucose and lactate were stable when chilled. Air bubbles interfered pO_2_ regardless of temperatures, whereas pCO_2_ increased significantly at 22°C after 30 min, and pH decreased after 90 min. Bicarbonate, sO_2_, O_2_Hb, sodium, and potassium were the unaffected parameters.

**Conclusions:**

Correct BGA results are essential, and arterial sample is precious. Therefore, if immediate analysis cannot be performed, up to one hour, syringes stored at room temperature will give reliable results when care is taken to minimize air within the blood gas specimen.

## Introduction

Arterial blood gas analysis (BGA) is an essential test used for years to provide precise assessment of oxygenation, ventilation, and acid-base status of critically ill patients. Results have a crucial role in monitoring patients with serial measurements, as even small variations can be of great importance due to the low biological variation of significant blood gas parameters (*1*-*3*).

Arterial blood gas analysis differs from other biochemical testings for providing such vital information in such a short time. Therefore, point of care devices are highly requested in emergency and intensive care units. However, fast results do not guarantee the correct results, as the devices out of laboratory control causes undetected errors (*3*, *4*). On the other hand, collecting and studying samples in the core laboratory brings along transportation caused interferences, such as clots and changed parameters due to prolonged storage, the pneumatic tube transport system caused air contamination, all of which shadows the value of the BGA (*5*, *6*).

According to the previous literatures, the blood gas specimens are among the most rejected samples which results in resampling, delay in analysis, and increased costs (*7*-*9*). It is still unknown under which conditions the stored samples maintain their stability or which samples can no longer give correct results. The diversity and inadequacy of the studies on this subject are also confusing and there are still reports that it is the right practice to transport the samples on ice. In addition to the variability of the ambient conditions, the syringe material used should be tested by the laboratory used, and poor quality devices should be eliminated (*10*, *11*).

Years ago, the glass syringes were replaced with the plastic ones for being safe and cheap. However, the permeability limits the usefulness of the plastic syringes instead of the less permeable glass ones that affects the results even beyond vital limits (*12*-*14*). We therefore tested the effect of storage temperature and time delay on blood gas stability. A subgroup analysis was performed to detect the effect of air on results during storage.

## Materials and methods

### Subjects

This experimental study was conducted in the intensive care unit (ICU) of the Izmir Atatürk Research and Training Hospital in an eight-month period. For the study, arterial blood samples were collected from 20 patients admitted to the same ICU, for whom daily arterial blood gas is required. The blood was obtained from their intra arterial catheter. We used 2 mL (electroliquid balanced 100 international unit dry lithium heparin) plastic syringes with gasket (Reference number: GNJ-S-2.5, Genject, Ankara, Turkey).

This study protocol was approved by the Interventional Clinical Studies Institutional Review Board (Reference number: 58/2018) of Izmir Katip Çelebi University, Turkey. The study design complies with the Declaration of Helsinki ethical standards. The legal guardian of each patient was informed about the procedure by the physician of the ICU, the co-author of the study. The written informed consents for the involvement in the study were signed by their legal guardian prior to blood collection.

### Methods

A total of 10 plastic syringes of arterial blood was collected, 2 mL of each sample. Any visible air bubble in the sample was expelled carefully. Syringes were also checked for clots. All were inverted gently up and down, rolled between the palms before the analysis.

For the purpose of the study, syringes were divided into two groups to stand in two different temperatures (in the refrigerator (4 °C) and at room temperature (22 °C)), and to contaminate with air. Following immediate analysis for baseline, syringes were stored at 4 °C or at 22 °C, then studied after 30, 60, 90, 120 minutes. A subgroup analysis was performed to detect the effect of air on results during storage.

To contaminate with air, we pulled down the plunger to force the room air inside the syringe. Eight samples of each subject were reconstituted as 2 mL blood with 0.5 mL air. Each time point, one of the contaminated samples was studied. For the non-contaminated samples, analyses were performed with the same samples stored at two different temperatures. The temperatures of the room (ICU) and the refrigerator were continuously monitored using data loggers.

Samples were analysed for pH, partial pressure of oxygen (pO_2_), carbon dioxide (pCO_2_), hemoglobin oxygen saturation (sO_2_), bicarbonate (HCO_3_^-^), lactate, p50 (oxygen tension at 50% hemoglobin saturation), O_2_Hb (oxyhemoglobin), glucose, sodium, and potassium. Bicarbonate and p50 were calculated by blood gas analyser (bicarbonate from Henderson-Hasselbalch equation, p50 from sO_2_ and pO_2_)) (*15*, *16*). The analyser we used (ABL800 Flex (Radiometer, Copenhagen, Denmark)) has a co-oximetry module based on a multiwavelength spectrophotometric optical system that measures total hemoglobin concentration, O_2_ saturation, and Hb fractions such as oxyhemoglobin (O_2_Hb), deoxyhemoglobin (HHb), methemoglobin (metHb), carboxyhemoglobin (COHb) in a blood sample through the absorption spectra of the given analytes. The spectrophotometer is connected *via* an optical fiber to a combined hemolyzer and measuring chamber (*16*). In this study, sO_2_ and O_2_Hb were measured by co-oximetry. The methods the analyser used are tabulated in Table 1.

**Table 1:** Methods, quality control assigned values, and within-run imprecision results for blood gas parameters

**Parameter**	**Method**	**Level**	**Mean**	**CV (%)**	**Ricos desirable (%)**
pH	Potentiometry	1	7.089	0.08	0.1
		2	7.395	0.03	0.1
pCO_2_, kPa	Potentiometry	1	9.44	2.17	2.4
		2	5.49	0.94	2.4
pO_2_, kPa	Amperometry	1	20.4	1.36	-
		2	14.9	1.37	-
SO_2_, %	Spectrophotometry	1	50	0.13	3.8
		2	97	0.32	3.8
O_2_Hb, %	Spectrophotometry	1	44.5	0.2	-
		2	92.4	0.2	-
p50, mmHg	Calculated	-	-	-	-
HCO_3_^-^, mmol/L	Calculated	1	23.9	1.26	2
		2	24.4	0.48	2
Na, mmol/L	Potentiometry	1	161	0.3	0.3
		2	140	0.3	0.3
K, mmol/L	Potentiometry	1	1.8	1.6	2.3
		2	3.8	1.3	2.3
Glu, mmol/L	Amperometry	1	1.6	2.6	2.8
		2	5.7	2.3	2.8
Lactate, mmol/L	Amperometry	1	4.5	2.64	13.6
		2	1.6	0	13.6
Radiometer, Qualicheck 5+ Level 1 and 2 were used for within study CV’s and were calculated by analysing blood gas parameters in 10 sequential runs. Bicarbonate and p50 were calculated by the analyser (16). Estimated CV’s were compared with the desirable quality specifications derived from Ricos *et al.* (17). CV% – coefficient of variation. Glu – glucose. HCO_3_- – bicarbonate. K – potassium. Na – sodium. O_2_Hb – oxyhemoglobin. pCO2 – partial pressure of carbon dioxide. pO2 – partial pressure of oxygen. p50 – oxygen tension at 50% hemoglobin saturation. sO2 – oxygen saturation. Level-1,2 – Quality control levels 1 and 2.					

The analytical performance of the blood gas analyser was evaluated. The within-run imprecision was tested through the measurement of the quality controls (Qualicheck 5+) at two levels, supplied by Radiometer, in 10 sequential runs. The internal quality control was run daily during the study period. Same analyser in the ICU was used by the same laboratory practitioner.

### Statistical analysis

Coefficients of variation (CV) of each parameter at each level were calculated with the formula CV% = standard deviation (SD)/Mean. The obtained precision estimates were compared against the preset desirable quality specifications for imprecision by Ricos *et al.* (*17*).

For bias estimation, the result of each parameter was compared with the baseline. Percentage deviations were calculated with the equation ((C_x_ - C_B_)/C_B_) x 100, where C_B_ is the baseline value and C_X_ the mean or median of the experimented sample.

The deviations were compared against the desirable quality specifications estimated by Ricos *et al.* and the Royal College of Pathologists of Australasia (RCPA) for allowable total error (*17*, *18*).

Statistical analyses were performed with paired samples *t*-test or Wilcoxon signed rank test, according to their distributions by using statistical package for Windows, Version 21.0, SPSS Inc. (Chicago, USA). A *P* value < 0.05 was considered statistically significant.

## Results

The CV’s calculated are presented in Table 1. The imprecisions were all within the estimated data by Ricos *et al.* (*17*). The data for pO_2_ and sO_2_ were not available in the preset criteria by Ricos *et al.*, for which the estimated CV’s were < 1.4 and < 4, respectively.

Arterial blood gas results related to the time and temperature changes are shown in Table 2.

**Table 2:** Evaluation of the analytical deviations in various storage conditions

**Parameter**	**Condition**	**Baseline**	**30 min** **(4°C)**	**60 min** **(4°C)**	**90 min** **(4°C)**	**120 min** **(4°C)**	**30 min** **(22°C)**	**60 min** **(22°C)**	**90 min** **(22°C)**	**120 min** **(22°C)**	**aTE (%)**
pH	w/o air	7.44 ± 0.06	7.43 ± 0.06	7.42 ± 0.06	7.42 ± 0.05	7.43 ± 0.06	7.43 ± 0.06	7.42 ± 0.06	7.41 ± 0.06	7.40 ± 0.07	
			-0.1%	-0.2%	-0.2%	-0.1%	-0.1%	-0.2%	-0.3%	-0.5%	0.5% / ± 0.04*
			P = 0.001	P < 0.001	P < 0.001	P = 0.004	P = 0.001	P < 0.001	P < 0.001	P < 0.001	
	with air	7.44 ± 0.06	7.43 ± 0.05	7.42 ± 0.05	7.42 ± 0.05	7.42 ± 0.06	7.43 ± 0.06	7.42 ± 0.06	7.41 ± 0.06	7.40 ± 0.06	
			-0.1%	-0.2%	-0.2%	-0.2%	-0.1%	-0.2%	-0.4%	-0.5%	
			P = 0.003	P = 0.003	P = 0.001	P = 0.001	P = 0.139	P < 0.001	P < 0.001	P < 0.001	
pCO_2_, kPa	w/o air	5.60 ± 1.11	5.84±1.18	5.95±1.28	5.86±1.19	5.79±1.18	5.75±1.16	5.88±1.29	5.97±1.29	6.04±1.44	
			4.3%	6.4%	4.8%	3.5%	2.8%	5.1%	6.7%	7.9%	5.7%^†^
			P < 0.001	P < 0.001	P = 0.001	P = 0.015	P < 0.001	P = 0.013	P = 0.005	P = 0.009	
	with air	5.60 ± 1.11	5.86 ± 0.98	5.90 ± 1.11	5.83 ± 1.29	5.87 ± 1.15	5.72 ± 1.08	5.93 ± 1.19	6.05 ± 1.21	6.14 ± 1.20	
			4.7%	5.5%	4.2%	4.9%	2.2%	6.0%	8.1%	9.8%	
			P = 0.004	P < 0.001	P = 0.068	P < 0.001	P = 0.011	P = 0.002	P < 0.001	P < 0.001	
pO_2_, kPa	w/o air	14.0 ± 3.29	15.1±3.36	16.4±3.65	16.9±3.91	17.8±4.27	14.2±3.01	14.0±2.93	13.5±3.01	13.6±2.84	
			7.3%	17.0%	20.7%	26.7%	0.9%	-0.7%	-3.8%	-3.2%	5%*
			P = 0.001	P < 0.001	P < 0.001	P < 0.001	P = 0.722	P = 0.843	P = 0.400	P = 0.495	
	with air	14.0 ± 3.29	19.5 ± 4.25	20.6 ± 4.25	21.2 ± 4.53	21.8 ± 4.61	17.3 ± 3.72	16.9 ± 3.82	16.6 ± 3.73	16.2 ± 3.11	
			38.7%	47.0%	51.1%	55.4%	23.0%	20.7%	17.9%	15.2%	
			P < 0.001	P < 0.001	P < 0.001	P < 0.001	P < 0.001	P < 0.001	P = 0.001	P = 0.003	
sO_2_, %	w/o air	98.4 (80.4-99.5)	98.1 (82.4-99.1)	98.2 (82.9-99.4)	98.5 (84.4-99.3)	98.7 (85.3-99.6)	98.0 (80.6-99.8)	97.9 (80.9-99.5)	97.8 (81.4-99.2)	98.1 (81.8-99.5)	
			-0.3%	-0.2%	0.2%	0.4%	-0.4%	-0.5%	-0.6%	-0.3%	4%*
			P = 0.698	P = 0.057	P = 0.005	P = 0.001	P = 0.203	P = 0.398	P = 0.421	P = 0.984	
	with air	98.4 (80.4-99.5)	98.9 (89.0-99.5)	99.1 (89.7-99.7)	99.2 (89.7-99.6)	99.2 (91.6-99.7)	98.7 (85.9-99.4)	98.5 (86.6-99.5)	98.6 (86.9-99.6)	98.3 (87.7-99.6)	
			0.6%	0.7%	0.8%	0.8%	0.3%	0.2%	0.3%	-0.1%	
			P < 0.001	P < 0.001	P < 0.001	P < 0.001	P = 0.001	P = 0.029	P = 0.064	P = 0.067	
O_2_Hb, %	w/o air	96.3 (78.1-97.5)	95.8 (80.1-97.4)	96.0 (80.7-98.3)	96.2 (82.0-97.4)	96.4 (82.9-97.6)	95.6 (78.4-97.3)	95.5 (78.9-98.0)	95.6 (79.2-97.1)	95.6 (79.4-97.8)	4%*
			-0.5%	-0.3%	-0.1%	0.1%	-0.7%	-0.8%	-0.7%	-0.7%	
			P = 0.943	P = 0.099	P = 0.073	P = 0.012	P = 0.205	P = 0.408	P = 0.211	P = 0.559	
	with air	96.3 (78.1-97.5)	96.7 (86.4-98.0)	96.7 (87.2-98.1)	96.9 (87.4-97.7)	96.9 (89.1-97.8)	96.3 (83.6-97.6)	95.8 (84.2-97.5)	96.1 (84.6-98.0)	96.0 (85.3-97.5)	
			0.4%	0.5%	0.6%	0.7%	0	-0.5%	-0.2%	-0.3%	
			P = 0.002	P = 0.002	P = 0.001	P = 0.001	P = 0.001	P = 0.107	P = 0.104	P = 0.210	
P50, mmHg	w/o air	25.2 ± 1.97	25.7 ± 1.84	25.9 ± 1.74	25.7 ± 1.49	25.7 ± 1.59	25.8 ± 1.81	26.2 ± 2,23	26.3 ± 1,76	26.7 ± 2.48	-
			1.8%	2.6%	1.9%	2.0%	2.1%	3.8%	4.2%	5.8%	
			P = 0.029	P = 0.011	P = 0.014	P = 0.060	P = 0.044	P = 0.026	P = 0.009	P = 0.007	
	with air	25.2 ± 1.97	25.7 ± 1.53	25.8 ± 1.41	25.9 ± 1.38	25.8 ± 1.52	25.6 ± 1.81	26.1 ± 1.80	26.6 ± 2.09	26.7 ± 2.12	
			1.9%	2.3%	2.5%	2.3%	1.3%	2.9%	5.4%	5.9%	
			P = 0.064	P = 0.025	P = 0.030	P = 0.050	P = 0.030	P = 0.002	P < 0.001	P = 0.005	
HCO_3_^-^, mmol/L	w/o air	28.0 ± 5.44	28.3 ± 5.69	28.3 ± 5.48	28.2 ± 5.52	28.0 ± 5.46	27.9 ± 5.36	27.8 ± 5.49	27.6 ± 5.42	27.2 ± 5.58	
			1.3%	1.2%	0.8%	0.3%	-0.3%	-0.4%	-1.2%	-2.8%	4.9%^†^
			P = 0.016	P = 0.118	P = 0.227	P = 0.673	P = 0.599	P = 0.552	P = 0.038	P < 0.001	
	with air	28.0 ± 5.44	28.2 ± 5.55	28.4 ± 5.52	27.9 ± 5.94	28.1 ± 5.48	28.1 ± 5.66	28.0 ± 5.48	27.6 ± 5.50	27.4 ± 5.47	
			1.0%	1.6%	-0.1%	0.6%	0.5%	0.1%	-1.1%	-2.1%	
			P = 0.016	P = 0.042	P = 0.913	P = 0.364	P = 0.362	P = 0.853	P = 0.039	P < 0.001	
Na, mmol/L	w/o air	142.6 ± 7.3	142.7 ± 7.6	142.0 ± 7.9	142.2 ± 7.4	141.6 ± 8.0	142.8 ± 7.2	142.2 ± 8.3	142.3 ± 7.5	142.2 ± 7.8	
			0.07%	-0.42%	-0.28%	-0.70%	0.14%	-0.28%	-0.21%	-0.28%	0.73%^†^
			P = 0.906	P = 0.305	P = 0.504	P = 0.156	P = 0.330	P = 0.453	P = 0.605	P = 0.487	
	with air	142.6 ± 7.3	143.0 ± 7.7	141.7 ± 7.7	142.1 ± 7.6	141.6 ± 7.8	143.0 ± 7.4	142.3 ± 7.8	142.7 ± 7.4	142.2 ± 7.9	
			0.28%	-0.63%	-0.35%	-0.70%	0.28%	-0.21%	0.07%	-0.28%	
			P = 0.185	P = 0.137	P = 0.409	P = 0.167	P = 0.090	P = 0.603	P = 0.934	P = 0.530	
K, mmol/L	w/o air	4.09 ± 1.04	4.09 ± 1.06	4.13 ± 1.04	4.17 ± 1.04	4.21 ± 1.03	4.05 ± 1.04	4.02 ± 1.05	3.99 ± 1.05	4.02 ± 1.03	
			0.1%	1.1%	2.0%	3.1%	-1.0%	-1.6%	-2.5%	-1.7%	5.6%^†^
			P = 0.577	P = 0.035	P = 0.006	P < 0.001	P = 0.002	P = 0.019	P = 0.027	P = 0.012	
	with air	4.09 ± 1.04	4.12 ± 1.06	4.13 ± 1.04	4.18 ± 1.05	4.25 ± 1.02	4.06 ± 1.06	3.99 ± 1.06	4.00 ± 1.07	4.01 ± 1.06	
			0.9%	1.1%	2.3%	3.9%	-0.6%	-2.2%	-2.1%	-1.8%	
			P = 0.015	P = 0.083	P = 0.001	P < 0.001	P = 0.030	P = 0.005	P = 0.006	P = 0.012	
Glu, mmol/L	w/o air	9.08 ± 4.04	8.95 ± 3.84	8.98 ± 3.90	8.91 ± 4.01	8.83 ± 4.04	8.98 ± 4.04	8.76 ± 4.05	8.57 ± 4.20	8.38 ± 4.34	
			-1.4%	-1.1%	-1.8%	-2.8%	-1.4%	-3.5%	-5.6%	-7.7%	6.96%^†^
			P = 0.003	P = 0.004	P < 0.001	P < 0.001	P = 0.013	P = 0.002	P < 0.001	P < 0.001	
	with air	9.08 ± 4.04	8.82 ± 3.76	8.81 ± 3.74	8.81 ± 3.95	8.78 ± 3.95	8.79 ± 3.92	8.60 ± 3.92	8.58 ± 4.15	8.44 ± 4.10	
			-2.8%	-2.9%	-3.0%	-3.3%	-3.2%	-5.3%	-5.5%	-7.0%	
			P = 0.003	P = 0.004	P < 0.001	P < 0.001	P < 0.001	P < 0.001	P < 0.001	P < 0.001	
Lactate, mmol/L	w/o air	1.97 ± 1.86	2.11 ± 1.70	2.20 ± 1.67	2.25 ± 1.65	2.34 ± 1.59	2.24 ± 1.78	2.52 ± 1.73	2.78 ± 1.71	3.07 ± 1.75	
			6.9%	11.4%	14.0%	18.5%	13.7%	27.9%	41.1%	55.8%	30.4%^†^
			P = 0.003	P = 0.003	P < 0.001	P < 0.001	P < 0.001	P < 0.001	P < 0.001	P < 0.001	
	with air	1.97±1.86	2.14 ± 1.67	2.19 ± 1.61	2.23 ± 1.68	2.29 ± 1.62	2.27 ± 1.68	2.47 ± 1.63	2.72 ± 1.66	2.93 ± 1.56	
			8.6%	10.9%	13.2%	16.0%	15.2%	25.4%	38.1%	48.7%	
			P = 0.003	P = 0.003	P < 0.001	P < 0.001	P < 0.001	P < 0.001	P < 0.001	P < 0.001	
*aTE – allowable total error by Royal College of Pathologists of Australasia (RCPA) (18). ^†^aTE – desirable specifications for allowable total error by Ricos *et al.* (17). COHb – carboxyhemoglobin. Glu – glucose. HCO_3_^-^ – bicarbonate. K – potassium. Na – sodium. O_2_Hb – oxyhemoglobin. pCO2 – partial pressure of carbon dioxide. pO2 – partial pressure of oxygen. p50 – oxygen tension at 50% hemoglobin saturation. sO2 – oxygen saturation. w/o – without air.											

### Without air bubbles

At room temperature, statistically significant decreases were observed for pH, glucose, potassium, and HCO_3_^-^, whereas statistically significant increases were found for pCO_2_, lactate, and p50. At 4 *°C*, statistically significant decreases were detected for pH, and glucose, whereas statistically significant increases were found for pO_2_, pCO_2_, lactate, potassium, HCO_3_^-^, sO_2_, O_2_Hb, and p50. Clinically significant biases were observed for pH, pCO_2_, lactate, and glucose at room temperature, for pO_2_ and pCO_2_ at 4 *°C* (Table 2).

### With air bubbles

Air bubbles interfered pO_2_ quickly. In samples stored at room temperature, statistically significant decreases were observed for pH, HCO_3_^-^, glucose, and potassium, whereas statistically significant increases were found for pO_2_, pCO_2_, sO_2_, lactate, and p50. In samples stored in the refrigerator, statistically significant decreases were observed for pH and glucose, whereas statistically significant increases were observed for pO_2_, pCO_2_, sO_2_, O_2_Hb, HCO_3_^-^, lactate, potassium, and p50. Clinically significant biases were observed for pH, pO2, pCO2, lactate, and glucose at room temperature, for pO_2_ at 4 *°C* (Table 2).

## Discussion

Changes in blood gas concentrations during storage have been evaluated in many studies under various conditions, hence resulted in different suggestions (*19*-*21*). According to our findings, BGA results were clinically acceptable in samples stored at room temperature for up to one hour, unexpectedly. However, contamination with air altered blood gases, especially the pO_2_, as expected.

The practice of keeping glass syringes on ice is an old practice to slow down the metabolism and is no longer recommended for plastic syringes (*1*, *3*). The rate of cellular metabolism is much faster in patients with very high leukocytes or platelet counts (*3*, *22*). The cells in the whole blood use glucose and O_2_, produce CO_2_, hence decrease the pH and increase lactate due to continued glycolysis. In this study, we used fresh blood samples to demonstrate cell metabolism. And we kept the syringes in the refrigerator in order to ensure homogeneous cooling at the specified degree. The small oxygen molecule (O_2_) is more vulnerable than larger CO_2_ molecules to diffuse across the plastic wall (*13*). The pores of the plastic syringe wall enlarge in the cold due to molecular contraction (*23*, *24*). Cooling the samples causes an increase in Hb’s affinity for oxygen to bind to deoxyhemoglobin, which promotes greater exogenous oxygen flow (*24*). This might result in the release of oxygen from the Hb when the sample is heated to 37 °C while analysis causes falsely increased pO_2_ (*12*, *25*).

In a study by Pretto and Rochford, plastic and glass syringes were compared for their gas preservation capacity (*14*). Glass syringes were found to be superior to plastic ones in preserving blood. As they observed similar increases in pCO_2_ accompanying a decrease in pH in both syringe types, they considered that these changes were due to cellular metabolism rather than diffusion through the syringe wall. In this study, we observed increases in pCO_2_ over time with a corresponding decrease in pH, possibly due to ongoing cell metabolism.

In an old study by Mahoney *et al.*, 30 min delay in analysis of arterial blood in plastic syringes caused pO_2_ increases when iced (*21*). In the current study, at room temperature, pO_2_ stayed stable for 120 min, however, showed significant increases (from 7.3% to 26.7%) when chilled, beginning from 30 min. We can attribute the increases observed in the pO_2_ to the diffusion of oxygen through the plastic wall of the syringe and decreases to the continuing metabolism of the cells.

The lactic acid values are important, especially for critically ill patients which have been shown to correlate with the prognosis in the setting of sepsis (*26*). Under anaerobic conditions, lactate is the degradation product of the metabolism and in erythrocytes, glycolysis always terminates in lactate. Keeping the plastic syringes at low temperatures increases the gas permeability that causes O_2_ supply and limit the anaerobic metabolism hence minimizes lactate production. In this study, lactate increased more at room temperature with a concomitant acidosis, and with a corresponding pO_2_ decrease, and pCO_2_ increase. As expected, in cooled samples, both with the effect of slowed down of the metabolism and the air diffusion through the syringe wall, lactate production was lesser.

Hemoglobin oxygen saturation (sO_2_) indicates the percentage of the Hb binding sites that actually bind to oxygen molecules, and O_2_Hb indicates the oxygen carrying form of hemoglobin in relation to the total hemoglobin which is approximately equal to the oxygen saturation in healthy individuals (*15*). Oxygen saturation is an important variable in determining blood oxygen delivery in the body. In a study by Arbiol *et al.*, where they stored samples in either iced water, at 4-8 °C and at 25 °C with a baseline sO_2_ > 95%, the samples were found as stable for sO_2_ for over 60 min (*27*). Smajić *et al.* stored plastic syringes at room temperature for 60 min and observed significant decreases in sO_2_ (-1.24%) (*28*). In our study, the sO_2_ changes were clinically insignificant with a baseline > 98%. And no clinically meaningful deviations were observed for O_2_Hb.

The partial pressure of oxygen required to achieve 50% hemoglobin saturation is represented as p50, which reflects the hemoglobin oxygen affinity and tissue oxygenation. This parameter is calculated by the blood gas analyser from oxygen tension (pO_2_) and saturation (sO_2_). In state of acidosis, p50 increases. In this study, we observed increases in p50, decreases in pH, which can be explained again with the continuing cell metabolism.

Sophisticated new blood gas devices made it possible to measure electrolytes and glucose in a short time, along with BGA in emergency and ICU units. Serial blood gas measurements allow serial electrolyte and glucose monitoring. This helps physicians in the diagnosis and monitoring of the blood gas and electrolyte imbalances. In this study, sodium, potassium, and glucose gave clinically acceptable results, irrespective of the storage conditions.

As expected, samples contaminated with air interfered pO_2_ quickly, similar to previous studies, and was greater in cold (*29*, *30*). However, blood pH stayed stable for over 90 min in all conditions. The stability of pH might be explained by the tight control and buffer capacity of blood (*6*). Bicarbonate was stable over two hours, unexpectedly, similar to a study (*25*). In a study by Toffaletti and McDonnell, adding air showed different effects on pO_2_ depending on the initial pO_2_ values even with small amounts of air (20 and 40 µL*)* (*20*). The direction of the changes in pO_2_ was very different with very low (< 9 kPa) and very high (> 24 kPa) initial values.

As a limitation of our study, for the air contaminated samples, the direction of change would be different according to the initial pO_2_, however, we did not experiment such extreme values (*20*). Besides, the results we obtained were for a type of plastic syringe, therefore the ingredients of different types of the syringe wall might affect the permeability variously.

For good laboratory practices, the laboratory technicians and health personnel have to be educated about the importance of this hardly obtained test material (*1*, *4*). They have to be warned about the air bubbles that should be expelled after withdrawing the sample as soon as possible and to transport without cooling samples.

In conclusion, correct BGA results are essential, and the arterial sample is precious. Therefore, if immediate analysis cannot be performed, up to one hour, syringes stored at room temperature will give reliable results when care is taken to minimize air within the blood gas specimen.

## References

[1] Clinical and Laboratory Standards Institute (CLSI). Blood gas and pH analysis and related measurements, 2nd edition. CLSI Document C46-A2. Wayne,PA:CLSI,2009.

[2] Simundic AM. Pre-examination procedures in laboratory diagnostics: preanalytical aspects and their impact on the quality of medical laboratory results. In: Guder WG, Narayanan S, eds. Blood gases, ions and electrolytes. Berlin, München, Boston: De Gruyter; 2015. p. 292-297. 10.1515/9783110334043-034 10.1515/9783110334043-034

[3] Baird G. Preanalytical considerations in blood gas analysis. Biochem Med (Zagreb). 2013;23:19–27. 10.11613/BM.2013.005 23457763 PMC3900096

[4] Dukić L Kopčinović LM Dorotić A Baršić I. Blood gas testing and related measurements: National recommendations on behalf of the Croatian Society of Medical Biochemistry and Laboratory Medicine. Biochem Med (Zagreb). 2016;26:318–36. 10.11613/BM.2016.036 27812301 PMC5082214

[5] Victor Peter J Patole S Fleming JJ Selvakumar R Graham PL. Agreement between paired blood gas values in samples transported either by a pneumatic system or by human courier. Clin Chem Lab Med. 2011;49:1303–9. 10.1515/CCLM.2011.611 21619479

[6] Collinson PO John CM Gaze DC Ferrigan LF Cramp DG. Changes in blood gas samples produced by a pneumatic tube system. J Clin Pathol. 2002;55:105–7. 10.1136/jcp.55.2.105 11865003 PMC1769597

[7] Bonini P Plebani M Ceriotti F Rubboli F. Errors in laboratory medicine. Clin Chem. 2002;48:691–8. 10.1093/clinchem/48.5.691 11978595

[8] Simundic AM Lippi G. Preanalytical phase – a continuous challenge for laboratory professionals. Biochem Med (Zagreb). 2012;22:145–9. 10.11613/BM.2012.017 22838180 PMC4062337

[9] Atay A Demir L Cuhadar S Saglam G Unal H Aksun S Clinical biochemistry laboratory rejection rates due to various types of preanalytical errors. Biochem Med (Zagreb). 2014;24:376–82. 10.11613/BM.2014.040 25351356 PMC4210258

[10] Lima-Oliveira G Lippi G Salvagno GL Montagnana M Picheth G Guidi GC. Different manufacturers of syringes: a new source of variability in blood gas, acid-base balance and related laboratory test? Clin Biochem. 2012;45:683–7. 10.1016/j.clinbiochem.2012.03.007 22440459

[11] Küme T Şişman AR Solak A Tuglu B Çinkooglu B Çoker C. The effects of different syringe volume, needle size and sample volume on blood gas analysis in syringes washed with heparin. Biochem Med (Zagreb). 2012;22:189–201. 10.11613/BM.2012.022 22838185 PMC4062339

[12] Ruppel GL. Of time and temperature, plastic and glass: specimen handling in the blood-gas laboratory. Respir Care. 2006;51:717–8. 16800902

[13] Wiwanitkit V. Glass syringes are better than plastic for preserving arterial blood gas for oxygen partial pressure determination: an explanation based on nanomaterial composition. Int J Nanomedicine. 2006;1:223–4. 10.2147/nano.2006.1.2.223 17722540 PMC2426785

[14] Pretto JJ Rochford PD. Effects of sample storage time, temperature, and syringe type on blood gas tensions in samples with oxygen partial pressures. Thorax. 1994;49:610–2. 10.1136/thx.49.6.610 8016801 PMC474964

[15] Kaufman DA. Interpretation of arterial blood gases. Available from: http://www.thoracic.org/clinical/critical-care/clinical-education/abgs.php. Accessed 2014.

[16] ABL800 Flex Reference manual 2008. Available from: http://www.radiometer.com

[17] Ricós C Alvarez V Cava F Garcia-Lario JV Hernandez A Jimenez CV Current databases on biologic variation: pros, cons and progress. Scand J Clin Lab Invest. 1999;59:491–500. updated in 2014. 10.1080/00365519950185229 10667686

[18] RCPA. Allowable limits of performance for biochemistry. Available from: https://www.westgard.com/rcpa-biochemistry.htm. Accessed May 2017.

[19] Biswas CK Ramos JM Agroyannis B Kerr DN. Blood gas analysis: effect of air bubbles in syringe and delay in estimation. Br Med J (Clin Res Ed). 1982;284:923–7. 10.1136/bmj.284.6320.923 6802352 PMC1496510

[20] Toffaletti JG, McDonnell EH. Effect of small air bubbles on changes in blood pO2 and blood gas parameters: calculated vs. measured effects. Available from: http://acutecaretesting.org. Accessed July 2012.

[21] Mahoney JJ Harvey J Wong R Van Kessel A. Changes in oxygen measurements when whole blood is stored in iced plastic or glass syringes. Clin Chem. 1991;37:1244–8. 10.1093/clinchem/37.7.1244 1823532

[22] Grzych G Roland E Beauvais D Maboudou P Lippi G. Leukocytosis interference in clinical chemistry: Shall we still interpret test results without hematological data? J Med Biochem. 2020;39:66–71. 10.2478/jomb-2019-0005 32549780 PMC7282239

[23] Knowles TP Mullin RA Hunter JA Douce FH. Effects of syringe material, sample storage time, and temperature on blood gases and oxygen saturation in arterialized human blood samples. Respir Care. 2006;51:732–6. 16800906

[24] Beaulieu M Lapointe Y Vinet B. Stability of pO2, pCO2, and pH in fresh blood samples stored in a plastic syringe with low heparin in relation to various blood-gas and hematological parameters. Clin Biochem. 1999;32:101–7. 10.1016/S0009-9120(98)00098-8 10211625

[25] Mohammadhoseini E Safavi E Seifi S Seifirad S Firoozbakhsh S Peiman S. Effect of sample storage temperature and time delay on blood gases, bicarbonate and pH in human arterial blood samples. Iran Red Crescent Med J. 2015;17:e13577. 10.5812/ircmj.13577 26019892 PMC4441774

[26] Garcia-Alvarez M Marik P Bellomo R. Sepsis-associated hyperlactatemia. Crit Care. 2014;18:503. 10.1186/s13054-014-0503-3 25394679 PMC4421917

[27] Arbiol-Roca A Imperiali CE Dot-Bach D Valero-Politi J Dastis-Arias M. Stability of pH, blood gas partial pressure, hemoglobin oxygen saturation fraction, and lactate concentration. Ann Lab Med. 2020;40:448–56. 10.3343/alm.2020.40.6.448 32539300 PMC7295962

[28] Smajić J Kadić D Hasić S Serdarević N. Effects of post-sampling analysis time, type of blood samples and collection tubes on values of blood gas testing. Med Glas (Zenica). 2015;12:108–12. 26276646 10.17392/823-15

[29] Woolley A Hickling K. Errors in measuring blood gases in the intensive care unit: effect of delay in estimation. J Crit Care. 2003;18:31–7. 10.1053/jcrc.2003.YJCRC7 12640611

[30] O’Connor TM Barry PJ Jahangir A Finn C Buckley BM El-Gammal A. Comparison of arterial and venous blood gases and the effects of analysis delay and air contamination on arterial samples in patients with chronic obstructive pulmonary disease and healthy controls. Respiration. 2011;81:18–25. 10.1159/000281879 20134147

